# Henipavirus-related Sequences in Fruit Bat Bushmeat, Republic of Congo

**DOI:** 10.3201/eid1809.111607

**Published:** 2012-09

**Authors:** Sabrina Weiss, Kathrin Nowak, Jakob Fahr, Gudrun Wibbelt, Jean-Vivien Mombouli, Henri-Joseph Parra, Nathan D. Wolfe, Bradley S. Schneider, Fabian H. Leendertz

**Affiliations:** Robert Koch-Institut, Berlin, Germany (S. Weiss, K. Nowak, F.H. Leendertz);; Technische Universität, Braunschweig, Germany (J. Fahr);; Leibniz Institute for Zoo and Wildlife Research, Berlin (G. Wibbelt);; Laboratoire National de Santé Publique, Brazzaville, Republic of Congo (J.-V. Mouli, H.-J. Parra);; and Global Viral Forecasting Initiative, San Francisco, California, USA (N.D. Wolfe, B.S. Schneider)

**Keywords:** Henipavirus, Paramyxoviridae, Africa, Chiroptera, *Eidolon helvum*, viruses, fruit bats

**To the Editor:** Bats are hosts for various emerging viruses, including the zoonotic paramyxoviruses Hendra virus and Nipah virus, which occur in Australia and Southeast Asia, respectively, and cause severe disease outbreaks among humans and livestock (*1*). Antibodies and henipavirus-related RNA have also been found in the straw-colored fruit bat, *Eidolon helvum*, in Ghana, West Africa (*2*,*3*). These bats are a chief protein source for humans in sub-Saharan Africa and are therefore targeted by hunters (*4*,*5*). This practice raises special concern about the risk for virus transmission from bats to humans.

To investigate the risk of zoonotic disease emergence through hunting and preparation and consumption of bats, in October 2009, we obtained animals from local hunters. This meat was destined to be sold at markets in downtown Brazzaville, Republic of Congo. All bats were *E. helvum*, one of the most frequently hunted and traded fruit bat species in Africa (*4*,*5*). According to hunters, bats were captured with nets in an area near the capital (4°22′40′′S, 15°06′27′′E) during the night and collected in the morning. Animals were maintained in cages until they were sold alive in the market. For this study, living bats were brought to the National Laboratory in Brazzaville.

All animals appeared clinically healthy on arrival at the laboratory. Animals were euthanized, and samples were stored immediately in RNA or later in liquid nitrogen; additional organ samples were transferred into a 10% buffered formalin solution. Neither macroscopic pathologic changes nor histopathologic evidence for viral infection was found. A total of 339 samples collected from 42 bats were tested for paramyxovirus RNA by PCR targeting L-gene sequences of respirovirus, morbillivirus, and henipavirus (*6*). Fifteen samples from 11 individual bats yielded a product of the expected size of 494 bp. These amplicons were cloned and underwent Sanger sequencing. Virus load in tissue samples, as determined by use of specific real-time PCR, ranged from 1.1 × 10^2^ to 3.4 × 10^4^ copies per piece (≈0.3 cm^3^). Four samples could not be quantified, probably because copy numbers were too low. Virus load in urine was 1.8 × 10^6^ per mL. For 4 of the 14 positive samples, we gathered additional sequence information by using pan-*Paramyxovirinae* primers targeting the most conserved genomic region (*6*). Sequencing of the cloned urine sample resulted in 2 distinct sequences for each fragment. Details regarding positive samples and dataset composition are found in the Technical Appendix.

In a phylogenetic tree, *Eidolon* paramyxovirus (EPMV) sequences are shown to form at least 3 distinct groups in the *Paramyxoviridae* family (Figure, panel A) and seem to be highly diverse compared with other paramyxovirus genera. At least 1 bat appeared to be infected with 2 different strains. Despite a geographic distance of >2,000 km among bats sampled, no spatial distinction was found between sequences from bats from Ghana and bats from the Republic of Congo. The same result can be seen when phylogenetic trees are built on the basis of the *Paromyxovirinae* fragment (Figure, panel B). In both trees, henipaviruses cluster in between EPMV sequences. Because EPMV and henipaviruses originate from fruit bats, this finding is not surprising. All animals in this study originate from a single locality just outside Brazzaville, the capital of the Republic of Congo. *E. helvum* bats are one of the most abundant species of fruit bats in sub-Saharan Africa; they roost in large colonies comprising up to 1 million animals. Bats in this species migrate up to 2,500 km per year, probably following seasonal changes in food availability (*7*). The diversity of distinct EPMV lineages recovered by this study at a single site, and the variable clustering with sequences retrieved from animals in Ghana, demonstrate that different strains are exchanged over large distances by migratory *E. helvum* bats.

**Figure:**
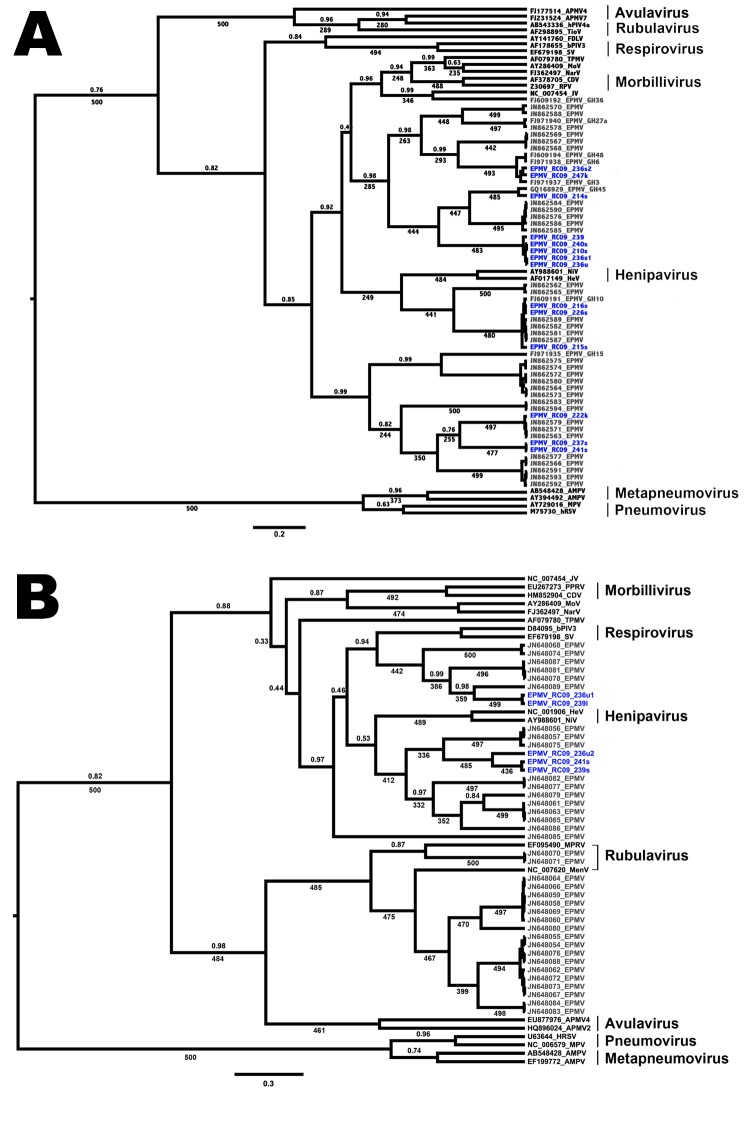
Phylogenetic trees showing the placement of *Eidolon* paramyxovirus (EPMV) sequences in the diversity of *Paramyxoviridae*, based on partial large gene sequences (384 nt and 474 nt, respectively) of the respirovirus, morbillivirus, and henipavirus fragment (A) and the *Paramyxovirinae* (PAR) fragment (B), Republic of Congo, 2009. EPMV sequences from Ghana are printed in gray, and novel EPMV sequences from the Republic of Congo are printed in blue. Trees were computed by using BEAST version 1.7.1 (http://beast.bio.ed.ac.uk/Main_Page) under the assumption of a relaxed, uncorrelated lognormal clock and the Yule process speciation model. Values given are posterior probabilities (above branches) and values resulting from nonparametric bootstrapping (below branches; 500 pseudoreplicates) after analysis in PhyML version 3.0 (http://www.atgc-montpellier.fr/phyml/). For better visibility, only posterior probabilities values <1 and bootstrap values >225 (45%) are indicated. Scale bar indicates substitutions per site. For detailed dataset composition and processing, see the Technical Appendix. Novel EPMV sequences are shown in blue and were deposited into GenBank under accession nos. HE647821–HE647839 and HE801055–HE801056. APMV, avian paramyxovirus; AMPV, avian metapneumovirus; bPIV, bovine parainfluenza virus; CDV, canine distemper virus; FDLV, fer-de-lance virus; HeV, Hendra virus; hPIV, human parainfluenza virus; HRSV, human respiratory syncytial virus; JV, J-virus; MenV, Menangle virus; MoV, Mossmann virus; MPRV, Mapuera virus; MPV, murine pneumonia virus; NarV, Nariva virus; NiV, Nipah virus; PPRV, peste-des-petits-ruminants virus; RPV, Rinderpest virus; SV, Sendai virus; TioV, Tioman virus; TPMV, Tupaia paramyxovirus.

Humans are exposed to these paramyxoviruses primarily by 2 mechanisms: 1) through bushmeat hunting (using nets or shotguns), handling, and consumption; and 2) through environmental contamination with bat excretions and saliva. *E. helvum* bats frequently roost in the middle of cities, and viral load in bat urine has been shown to be high. In Bangladesh, humans became infected with Nipah virus after consuming palm sap contaminated by bat urine and saliva (*8*). Infection of domestic pigs in Ghana (*9*) might also be a result of contact with bat excreta, which is especially troubling because pigs have acted as amplifying hosts in previous Nipah virus outbreaks in humans (*10*).

Despite the substantial exposure suggested by this study, to our knowledge, no human infection associated with bat paramyxoviruses has been reported in Africa, and elevated numbers of deaths have not been observed in bat hunters. Nevertheless, the existence of isolated cases cannot be excluded because underreporting is widespread, and many cases are undiagnosed. Additional studies on virus-host ecology, along with clinical surveys of exposed persons (hunters, vendors, cooks, etc.), are required to assess the zoonotic risk of these viruses and, ultimately, diminish the threat of a novel paramyxovirus entering and spreading in human populations.

Technical Appendix Details regarding positive samples and dataset composition.
