# Supplementary material for: Henipavirus-related Sequences in Fruit Bat Bushmeat, Republic of Congo
Source: Emerg Infect Dis. 2012 Sep;18(9):1536–7. doi: 10.3201/eid1809.111607 (PMC3437727; doi:10.3201/eid1809.111607)
Supplement: Technical Appendix — Details regarding positive samples and dataset composition. [file 11-1607-Techapp-s1.pdf]

Article DOI: http://dx.doi.org/10.3201/eid1809.111607

Henipavirus-related Sequences in Fruit Bat Bushmeat, Republic of Congo

Technical Appendix

Details Regarding Positive Samples and Dataset Composition

Animals obtained for this study were euthanized under anesthesia with xylazine and ketamine by cardiac exsanguination. Tissue samples were extracted by using the NucleoSpin RNA II Kit (Macherey-Nagel, Düren, Germany); throat swab specimens were dissolved in 500 µL of phosphate-buffered saline and extracted by using the QIAamp Viral RNA Mini Kit (QIAGEN, Hilden, Germany). The same kit was utilized for RNA extraction from urine. Fecal samples were extracted with the Stool DNA Extraction Kit (ROBOKLON, Berlin, Germany) in the presence of carrier RNA. RNA was reverse transcribed with random hexamer primers. The 339 samples, from 42 bats that were tested, included major organs of all animals (kidney, spleen, lung, small intestine). Liver samples were only available from 41, blood samples from 39, salivary gland of 1, throat swab specimens from 28, fecal samples from 31, and urine samples from 21 individual animals. Fifteen samples that tested positive with respirovirus, morbillivirus, and henipavirus (RES-MOR-HEN) PCR included 10 spleens, 3 kidneys, 1 liver, and 1 urine sample. Only 1 organ from each animal tested positive, except for 1 animal (RC09_Eid_239) which had viral sequences found in kidney, liver, and spleen. In 1 animal (RC09_Eid_236), spleen and urine samples tested positive. Five sequence-specific real-time PCRs were designed and used to re-screen spleen, kidney, and urine samples. Two spleen samples and 1 urine sample displayed additional positive results with very low copy numbers in 1 of 2 independent experiments. Because of low copy numbers and limitation of material, no sequences were derived for these samples. *Paramyxovirinae* (PAR) sequences were obtained for the positive liver and urine samples and for 2 positive spleen samples.

L-gene sequences for phylogenetic trees were downloaded from GenBank for all paramyxoviruses available on September 21, 2011, and reduced to unique sequences by using

the FaBox program (version 1.40) (*1*). From those, we assembled separate datasets for all paramyxovirus genera, which were aligned at the amino acid level by using the muscle algorithm as implemented in MEGA (version 5) (*2*). The best-fit model of nucleotide substitution was determined for all datasets using maximum likelihood–based Akaike information criterion scores (*2*). Maximum likelihood (ML) trees were generated with MEGA and, for each genus, the 2 sequences exhibiting the maximum patristic distances were determined with PATRISTIC (version 1) (*3*). Resulting sequences were used to build up final datasets, which additionally included all available paramyxovirus sequences from *Eidolon* bats and those not assigned to an existing genus. Final datasets were thus comprised of 74 and 59 sequences (RES-MOR-HEN and PAR fragment, respectively). These datasets were aligned at the amino acid level using the muscle algorithm as implemented in SeaView, version 4.2.12. (*4*). To increase alignment quality. we selected conserved blocks from protein alignments using the Gblocks server (*5*). Blocks were reported on the corresponding nucleotide alignments, which were manually edited for final refinement. Model selection on both resulting alignments was performed using the jModeltest program (version 0.1.1) (*6*) and resulted in selecting a general time reversible model with gamma site heterogeneity (+G) and invariant sites (+I) for both fragments.

Phylogenetic analyses were performed in both ML and Bayesian frameworks. ML analyses were performed using PhyML version 3.0 (*7*), and nearest-neighbor interchange (NNI) and subtree pruning and regrafting algorithms were applied for tree search. Branch robustness was assessed by performing nonparametric bootstrapping (500 pseudo-replicates). Bayesian analyses were performed using BEAST (version 1.7.1) (*8*) under the assumption of a relaxed, uncorrelated lognormal clock and the Yule process speciation model. Two independent runs of 62,000,000 generations were performed for the RES-MOR-HEN; 15,000,000 generations were performed for the PAR fragment. Trees and numerical values taken by all parameters were sampled every 1,000 generations. The Tracer program (version 1.5) (*9*) was used to check that individual runs had reached convergence, that independent runs converged on the same zones of parameter spaces, and that chain mixing was satisfactory (global effective sample size values >100 and >200 for RES-MOR-HEN and PAR, respectively). After removal of a visually conservative 10% burn-in period, tree samples were gathered into a single file using the LogCombiner program (version 1.7.1; distributed with BEAST), and the information was summarized onto the maximum clade credibility trees using the TreeAnnotator program (version

1.7.1; distributed with BEAST). Posterior probabilities were taken as a measure of branch robustness.

References

1. Villesen P. FaBox: an online toolbox for FASTA sequences. Mol Ecol Notes. 2007;7:965–8. http://dx.doi.org/10.1111/j.1471-8286.2007.01821.x

2. Tamura K, Peterson D, Peterson N, Stecher G, Nei M, Kumar S. MEGA5: Molecular Evolutionary Genetics Analysis using maximum likelihood, evolutionary distance, and maximum parsimony methods. Mol Biol Evol. 2011;28:2731–9. PubMed http://dx.doi.org/10.1093/molbev/msr121

3. Fourment M, Gibbs MJ. PATRISTIC: a program for calculating patristic distances and graphically comparing the components of genetic change. BMC Evol Biol. 2006;6:1. PubMed http://dx.doi.org/10.1186/1471-2148-6-1

4. Gouy M, Guindon S, Gascuel O. SeaView version 4: a multiplatform graphical user interface for sequence alignment and phylogenetic tree building. Mol Biol Evol. 2010;27:221–4. PubMed http://dx.doi.org/10.1093/molbev/msp259

5. Talavera G, Castresana J. Improvement of phylogenies after removing divergent and ambiguously aligned blocks from protein sequence alignments. Syst Biol. 2007;56:564–77. PubMed http://dx.doi.org/10.1080/10635150701472164

6. Posada D. jModelTest: phylogenetic model averaging. Mol Biol Evol. 2008;25:1253–6. PubMed http://dx.doi.org/10.1093/molbev/msn083

7. Guindon S, Dufayard J-F, Lefort V, Anisimova M, Hordijk W, Gascuel O. New algorithms and methods to estimate maximum-likelihood phylogenies: assessing the performance of PhyML 3.0. Syst Biol. 2010;59:307–21. PubMed http://dx.doi.org/10.1093/sysbio/syq010

8. Drummond AJ, Rambaut A. BEAST: Bayesian evolutionary analysis by sampling trees. BMC Evol Biol. 2007;7:214. PubMed http://dx.doi.org/10.1186/1471-2148-7-214

9. Rambaut A, Drummond AJ. Tracer v1.4. 2007. http://tree.bio.ed.ac.uk/software/tracer/
